# Influence of Early Stress on Social Abilities and Serotonergic Functions across Generations in Mice

**DOI:** 10.1371/journal.pone.0021842

**Published:** 2011-07-25

**Authors:** Tamara B. Franklin, Natacha Linder, Holger Russig, Beat Thöny, Isabelle M. Mansuy

**Affiliations:** 1 Brain Research Institute, Medical Faculty of the University of Zürich and Department of Biology, Swiss Federal Institute of Technology, Zürich, Switzerland; 2 Division of Clinical Chemistry and Biochemistry, University Children's Hospital, Zürich, Switzerland; University of North Dakota, United States of America

## Abstract

Exposure to adverse environments during early development is a known risk factor for several psychiatric conditions including antisocial behavior and personality disorders. Here, we induced social anxiety and altered social recognition memory in adult mice using unpredictable maternal separation and maternal stress during early postnatal life. We show that these social defects are not only pronounced in the animals directly subjected to stress, but are also transmitted to their offspring across two generations. The defects are associated with impaired serotonergic signaling, in particular, reduced 5HT1A receptor expression in the dorsal raphe nucleus, and increased serotonin level in a dorsal raphe projection area. These findings underscore the susceptibility of social behaviors and serotonergic pathways to early stress, and the persistence of their perturbation across generations.

## Introduction

Maternal interactions and maternal care during early life are essential for the development of appropriate social skills in adulthood in humans, primates and rodents [1], [2], [3], [4], [5], [6]. In humans, numerous clinical studies have reported that deficits in social behaviors have a high rate of inheritance and often run in families. Interestingly, the risk to develop social psychopathological symptoms is independently associated with parental psychopathology and with parental rearing, suggesting that both heritable and environmental factors jointly contribute to these symptoms [7], [8]. However, to date, there is no experimental evidence supporting this hypothesis. To study the impact of compromised early environment on social behaviors and its potential inheritance, we used a model of postnatal stress in the mouse. This model is based on unpredictable maternal separation combined with maternal stress (MSUS), a paradigm that perturbs maternal care provided to pups during the separation period, but not to their subsequent offspring [9]. MSUS was applied to primiparous C57Bl/6J females (F0) and their litters (F1) for two weeks starting after birth. The impact of the manipulation on social behaviors was further assessed on two following generations (F2 and F3) by breeding adult F1 males to wild-type C57Bl/6J females, then F2 males to wild-type females.

## Materials and Methods

### Animals

C57Bl/6J females and males (2½ months) were obtained from Elevage Janvier (Le Genest Saint Isle, France) and maintained in a temperature- and humidity-controlled facility on a 12h reversed light/dark cycle with food and water *ad libitum*. All procedures were carried out in accordance with the guidelines of the Veterinary Office of the Canton of Zurich, Switzerland, and approved by its Commission for Animal Research (210/2008). Maternal behaviors were monitored during the first two weeks after delivery. Once weaned, pups were reared in social groups (3–4 mice/cage) composed of animals subjected to the same treatment but from different dams to avoid litter effects. To produce a second and third generation, F1 control and MSUS males, or F2 control and MSUS males, were mated with naïve C57Bl6/J females. F2 and F3 offspring were weaned at PND21 and reared in mixed social groups similarly to F1.

### Maternal separation

Dams and litters were subjected to unpredictable maternal separation combined with unpredictable maternal stress (MSUS) for 3 hours daily from postnatal day 1 to 14 (PND 1–14) or left undisturbed apart from a cage change once a week (control) until weaning (PND21), as previously described [9]. Maternal stress consisted of either 20-min restraint in a Plexiglas tube or 5-min forced swim in cold water (18°C) applied unpredictably and randomly during separation. During separation, dams and pups were placed in separate clean cages with bedding and the dam's cage contained food and water. Dams and litters were placed close enough to have visual and olfactory contact. The timing of separation was unpredictable, but was always during the dark cycle. MSUS and control dams, and their litters had their cages changed on PND1, PND7, PND14, and PND21. Only dams giving birth within one week of each other were used, and litters with less than 4 pups were not used.

### Behavioral testing

In all cases, the experimenter was blind to treatment, and behaviors were monitored by direct observation, videotaping and videotracking (Ethovision, Noldus Information Technology). To avoid possible litter effects, pups from a minimum of 7 independent litters per treatment group (generated in the same experiment) were tested. All testing occurred under dim red light.

#### Investigation of same-sex conspecific

F1 males, and F2 and F3 males and females were placed in individual cages, identical to the homecage but containing a clear plexiglass barrier, and allowed 15-min habituation to the testing cage followed by 5-min habituation in the testing room. The test consisted of one 4-min exposure to a conspecific mouse of the same sex (3–4 weeks old). Conspecifics were placed behind the barrier that allowed visual and olfactory contact, but no direct physical contact. To assess possible reversal of social withdrawal by the selective serotonin 1A receptor (5HT1AR) agonist, 8-hydroxy-*N*,*N*-dipropyl-2-aminotetralin (8-OH-DPAT), F3 MSUS and control mice were injected with 15 µg/kg of 8-OH-DPAT i.p. 30 min prior to behavioral testing.

Time spent investigating the enclosure containing the conspecific was scored manually.

#### Social recognition memory

F1 male, and F2 and F3 female MSUS and control mice were placed in individual cages and allowed 15-min habituation. Cages were identical to the home cage, but contained a clear plexiglas barrier. The social recognition test consisted of two 4-min exposures to a conspecific mouse of the same sex (3–4 week old). Each exposure was separated by a 35-min retention interval and preceded by 5-min habituation to the testing room in the home cage. Conspecifics were placed behind the barrier that allowed visual and olfactory contact, but no direct physical contact. Time spent investigating the enclosure containing the conspecific was scored manually. Reduced investigation on 2^nd^ exposure (testing) compared to 1^st^ exposure (training) indicates proper social recognition memory. A discrimination ratio was determined such that 0.5 represents no difference in investigation time during testing and training (time spent investigating during testing/(time spent investigating during testing + time spent investigating during training). As control, olfactory recognition memory was tested in the same animals one week later, with a non-offensive scent (almond or vanilla) instead of an animal during training and testing.

#### Social defeat

Chronic social defeat (SD) was carried out as previously described [10]. C57Bl6/J male breeders screened for high aggressiveness (characterized by an attack latency of less than 3 min over 3 consecutive days) were used as resident aggressors. F2 MSUS and control males were used as intruders. Intruders were daily exposed to the same aggressor for 45 min over 14 days. They were first placed alone for 30 min in a mesh wire protective cover (l: 14 cm; w: 17 cm; h: 9 cm) inside the resident aggressor home cage (l: 36 cm; w: 18 cm, h: 14 cm) and allowed unrestricted visual, auditory and olfactory exposure to the resident but no physical contact. The protective cover was then removed and physical confrontation with the resident aggressor was allowed for 15 min. Non-defeated intruders (non-SD) were subjected to the same procedure with no resident aggressor in the second session. Sucrose consumption (4%, 10 hours) was monitored once daily. Twenty-four hours after the last SD session, mice were tested for their avoidance of aggressor olfactory cues by being placed for 3 min in a novel arena (l: 45 cm, w: 45 cm, h: 40 cm) containing an empty mesh wire enclosure, under which an unfamiliar aggressor was previously placed for 10 min, similar to Tsankova et al. [11]. Time spent around the enclosure (up to 8 cm away) and in corner zones was recorded using a videotracking system (Noldus).

### 8-OH-DPAT receptor binding

#### Tissue preparation

F2 male mice were killed by cervical dislocation, brains were rapidly removed, washed briefly in ice cold 0.1 M PBS, then embedded in frozen section medium and stored at −80°C. Frozen coronal slices (20 µm) were prepared using a microtome-cryostat (Cryo-Star HH 560 M, Microm International, Germany), thaw-mounted onto glass slides, air-dried and stored at −80°C.

#### Receptor autoradiography

5HT1AR binding was measured by quantitative ligand binding autoradiography as described by Ase et al.[12], using the selective 5-HT1AR agonist [H3]8-OH-DPAT (Perkin Elmer) as radioactive ligand. Coronal brain slices mounted on glass slides were preincubated at 25°C for 15 min in 50 mM Tris-HCl buffer (pH 7.40), then incubated for 60 min in the same buffer with 2 nM [H3]8-OH-DPAT [13] (Perkin Elmer). Slides were washed twice in ice cold buffer for 10 min and 5 min, briefly dipped into distilled water then dried for 3 hours in a stream of cold air. Non-specific binding was determined on adjacent sections using the same main incubation solution containing 2 nM [H3]8-OH-DPAT and 10 µM serotonin. The slides were exposed to a tritium sensitive screen (Packard instrument) together with a [H3]-microscale (Amersham) for 1 week. After exposure, sections were Nissl stained with 2% cresyl violet and brain areas of interest were identified and outlined using a mouse brain atlas. Photographs of Nissl stained sections with outlined regions were then overlapped with corresponding phosphorimages in Photoshop CS2 (Adobe) and phosphorimages were quantified using OptiQuant 4.0 (Packard). Labeling values of brain regions were corrected for screen background and nonspecific binding. For each region, a mean DLU of the region of interest was measured, and a mean between 2–3 selected sections was calculated. Standard curves generated from [H3]-microscales were used to convert DLU values into femtomoles of ligand per milligram of tissue using the following equation [14]: X Ci/mg tritium tissue equivalent for ligand/Specific activity of ligand =  X M ligand/mg tissue.

The following planes were used for 8-OH-DPAT binding quantification: −4.36 periaqueductal grey (PAG; lateral PAG and dorsal PAG), dorsal raphe (DR) and median raphe (MR); −1.82, hippocampus (CA1, CA2/3, dentate gyrus (DG)), posterior thalamus (posterior thal. containing ventral posterolateral and posteromedial thalamic nuclei, and posterior thalamic nucleus) and medial thalamus (med thal. containing central medial thalamic nucleus, intermediodorsal thalamic nucleus, and mediodorsal thalamic nucleus); −0.82, anterior thalamus (ant. thal. containing ventral posterolateral and anterior thalamic nuclei, and anteromedial thalamic nuclei), and hypothalamus (anterior hypothalamic area (AHC) and paraventricular nucleus (PVN); and +0.74, cortex (cingulate cortex (Cg1/2) and motor cortex (M1/M2), and striatum (caudate putamen (CPu) and nucleus accumbens (NAcc)).

#### High-performance liquid chromatography (HPLC)

F2 MSUS and control males were killed by cervical dislocation and their brains were rapidly removed and flash frozen. Brains were partially thawed and frontal cortex (∼Bregma +1.5 – 0), and dorsal hippocampus (∼Bregma -1.2 – −2.5) were removed. Samples were homogenized at 4°C in HPLC homogenization buffer (50 mM Tris-HCl, pH = 7.5, 100 mM KCl, 1 mM EDTA, 1 mM DTT, 0.2 mM PMSF, 1 µM leupeptin, 1 µM pepstatin), and protein was quantified (Protein Assay, Bio-rad). The homogenate was acidified using 1 M HCl then filtered (Amicon Ultra Centrifugal Filters Ultracel-10K Membrane, Millipore). Serotonin was determined using HPLC according to a previously published method [15].

#### Statistical analyses

All behavioral results in F1, F2 and F3 control and MSUS mice, and 8-OH-DPAT binding in F2 control and MSUS mice were analyzed using unpaired t-tests, except social defeat data and 8-OH-DPAT reversal which were analyzed using 2×2 ANOVAs (stress treatment x social defeat treatment and stress treatment x 8-OH-DPAT administration, respectively) followed by Fisher's PLSD post-hoc, when appropriate. As a control for social recognition and olfactory memory tests, one-sample t-test was performed to ensure that learning occurred in control groups (expected value = 0.5). All analyzed data matched the requirements for parametric statistical tests. Significance was set at p<0.05 for all tests. Error bars represent SEM. *, p<0.05; **, p<0.01.

## Results

**Figure 1:**
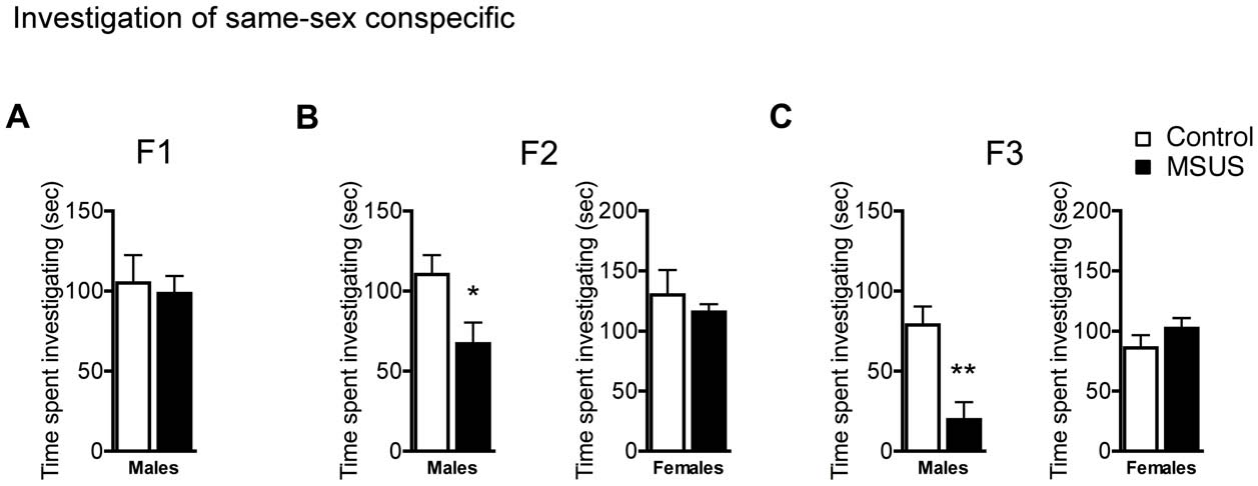
Abnormal sociability in F2 and F3 MSUS males. **a–c** Level of investigation of a same-sex unfamiliar conspecific in (**a**) F1 MSUS and control males, and (**b),** F2 and (**c**) F3 MSUS and control males and females (F1: ns; F2 male: t(13) = 2.38, p<0.05; F2 female: ns; F3 male: t(21) = 3.73, p<0.01; F3 female: ns).

**Figure 2:**
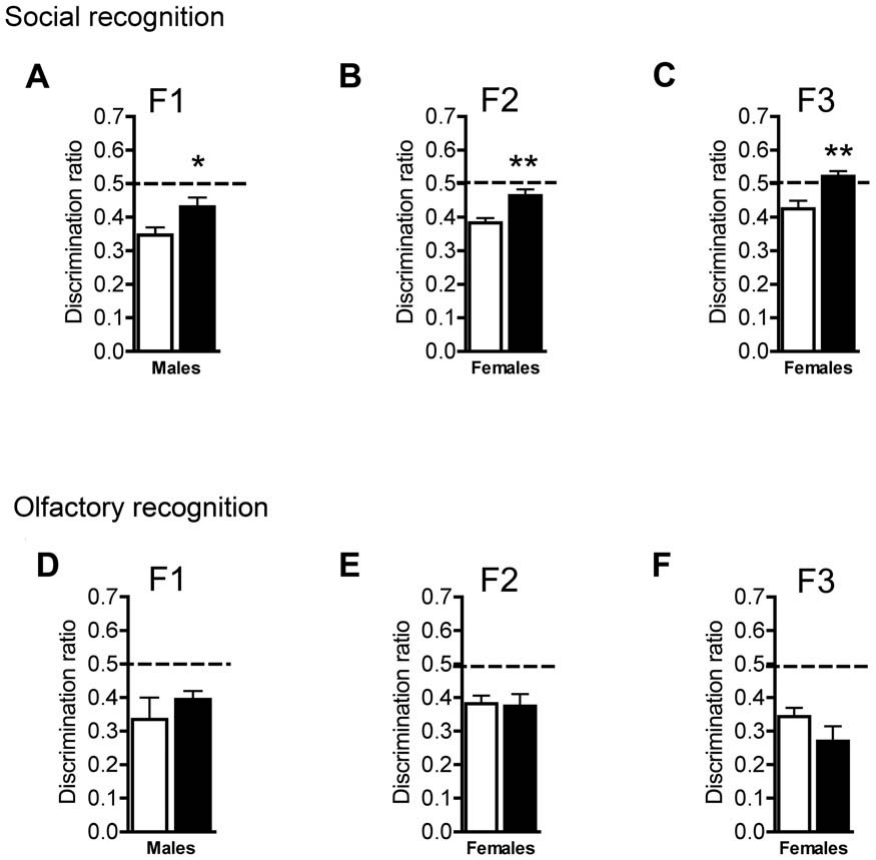
Abnormal social memory in F1, F2 and F3 MSUS mice. **a-c**, Social recognition discrimination ratio in (**a**) F1, (**b**) F2, and (**c**) F3 control and MSUS mice (F1: t(14) = 2.224, p<0.05; F2: t(14) = 3.43, p<0.01; F3: t(13) = 3.52, p<0.01). **d–f**, Olfactory memory discrimination ratio in (**d**) F1 MSUS and control males, and (**e**) F2, and (**f**) F3 MSUS and control females. One-sample t-test demonstrates significant decrease from expected value (0.5) in F1, F2, and F3 MSUS and control mice in the olfactory recognition test. *, p<0.05, **, p<0.01 as indicated by unpaired t-test, or Fisher's PLSD post-hoc where appropriate.

Since early stress has been implicated in antisocial behaviors [16], we examined whether social exploration was altered by MSUS in F1, F2 and F3 mice. We first assessed the animals' response to their 1^st^ exposure to a same-sex unfamiliar conspecific (non-threatening juvenile) that normally elicits high levels of social interaction and investigation of the conspecific. F1 MSUS and control males spent a comparable amount of time investigating their conspecific (Fig. 1a), however F2 and F3 MSUS males investigated their conspecific significantly less than controls, suggesting social anxiety or social withdrawal (Fig. 1b, c). This trait was specific to males since social investigation was not altered in F2 and F3 MSUS females (Fig. 1b, c). During the 2^nd^ exposure to the same conspecific, control animals investigated their conspecifics less than during the 1^st^ exposure, due to habituation resulting from appropriate social recognition memory. However, F1 MSUS males had no reduction, suggesting a deficit in social memory (Fig. 2a–c) [17]. Likewise, F2 and F3 MSUS females showed no decrease in investigation during 2^nd^ exposure to a conspecific. The deficit was specific to social memory since F1, F2, and F3 MSUS animals were able to form and retain a memory of olfactory cues not related to conspecifics similarly to controls (Fig. 2d–f). These results suggest a combination of social anxiety and impaired social memory in MSUS mice that is transmitted across generations. The profile of transmission was consistent across the 2^nd^ and 3^rd^ generations even though social anxiety was not clearly expressed in F1 MSUS males. Thus, these males appear to act as asymptomatic carriers, transmitting social anxiety to their F2 offspring, which themselves transmit this trait further to F3 offspring. This may result from the fact that F1 animals were exposed to MSUS only postnatally, while F2 offspring carry the effect transmitted from their father through both pre- and postnatal development, and therefore are more affected. The presence of these abnormal behaviors in the F2 and F3 MSUS generation suggests an epigenetic mechanism involving the germline.

**Figure 3:**
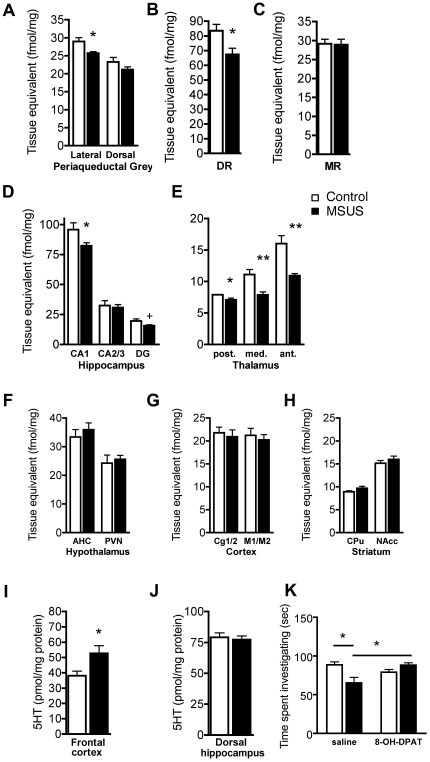
5HT1AR binding and serotonin level in the brain of MSUS offspring. **a–e,** 5HT1AR binding in (**a**) PAG, (**b**) DR, (**c**) MR, (**d**) hippocampus, (**e**) thalamus, (**f**) hypothalamus, (**g**) frontal cortex, and (**h**) striatum (lateral PAG: (t(8) = 2.79, p<0.05; DR: t(8) = 2.72, p<0.05; CA1: t(8) = 29.34, p<0.05; DG: t(8) = 2.26, p = 0.05); posterior thalamus: t(8) = 2.36, p<0.05; medial thalamus: t(8) = 3.67, p<0.01; anterior thalamus: t(8) = 3.81, p<0.01). Serotonin levels in (**i**) frontal cortex (t(16) = −2.54, p<0.05) and (**j**) dorsal hippocampus (t(16) = .43, ns) in MSUS and control males. (**k**) Effect of 8-OH-DPAT treatment on social exploration in MSUS and control males (F(1, 22) = 11.47, p<0.01). Abbreviations: Anterior hypothalamic area, AHC; anterior thalamus, ant. thal.; caudate putamen, CPu; cingulate cortex, Cg1/2; dentate gyrus, DG; dorsal raphe, DR; motor cortex, M1/M2; medial thalamus, med. thal.; median raphe, MR; nucleus accumbens, NAcc; paraventricular nucleus of the hypothalamus, PVN. periacqueductal grey, PAG; posterior thalamus, posterior thal. +, p = 0.05, *, p<0.05, **, p<0.01 as indicated by unpaired t-test, or Fisher's PLSD post-hoc when appropriate.

Early deficits in serotonin signaling via the serotonin (5HT) 1A receptor (5HT1AR) lead to heightened social anxiety in 5HT1AR knockout mice [18], and patients with social anxiety disorder have reduced 5HT1AR binding in the brain [19], [20], [21]. We examined whether 5HT1AR binding is altered by MSUS, in particular in the offspring of MSUS males, by using a radioligand with high affinity for the 5HT1AR, 8-OH-DPAT. Binding assays revealed that 8-OH-DPAT binding is reduced in several brain areas important for anxiety, stress and defense reactions in MSUS mice when compared to controls. These brain regions included the lateral, but not dorsal periaqueductal grey (PAG), the dorsal raphe (DR), but not median raphe (MR), CA1 and dentate gyrus (DG), but not CA2/3 subregions of the hippocampus, and several regions of the thalamus (Fig. 3a-e). Regions in the hypothalamus, cortex, and striatum were not affected (Fig. 3f–h). While 8-OH-DPAT binds 5HT1AR selectively, it can also bind other receptors such as the 5HT7R and the alpha1-adrenoreceptor, albeit with much lesser affinity [22], [23]. Thus, the reduced binding in F2 MSUS mice, although predominantly resulting from reduced 5HT1AR binding, may also reflect alterations in other receptor pathways.

Since 5HT1ARs in the DR and MR are autoreceptors that inhibit serotonergic projections to several brain areas [24], [25], we next examined whether serotonin release is increased in areas that receive DR projections in MSUS mice. To investigate this possibility, we measured the level of serotonin in the frontal cortex, a DR target area, and dorsal hippocampus, a MR target area, in F2 MSUS mice by high-performance liquid chromatography (HPLC). Consistent with the hypothesis, we found that the level of serotonin was increased in frontal cortex, but not in dorsal hippocampus, in F2 MSUS mice (Fig. 3i, j), suggesting a functional consequence of reduced 5HT1AR binding in DR. This finding significantly extends previous data demonstrating enhanced serotonin release following uncontrollable and chronic stress, and reduced autoreceptor activation in the DR [26], [27], [28], [29], [30], by newly showing that such functional alterations are persistent and can be transmitted across generations. To confirm that altered serotonergic signaling is also involved in social anxiety and its transmission to following generations in MSUS mice, we assessed the effect of the selective 5HT1AR agonist, 8-hydroxy-2-dipropylaminotetralin (8-OH-DPAT), on social behaviors. Acute administration of 8-OH-DPAT fully reversed the deficit in social interaction in F3 MSUS males, and normalized the time animals spent investigating their conspecific (Fig. 3k). This experiment was also performed in F3 females, but no conclusions could be drawn due to social avoidance induced by the injection itself in both control and MSUS mice (results not shown). However, the data in males suggest that similar molecular alterations involving reduced signaling via the 5HT1AR underlie the transmissible social anxiety observed in both F2 and F3 MSUS mice.

**Figure 4:**
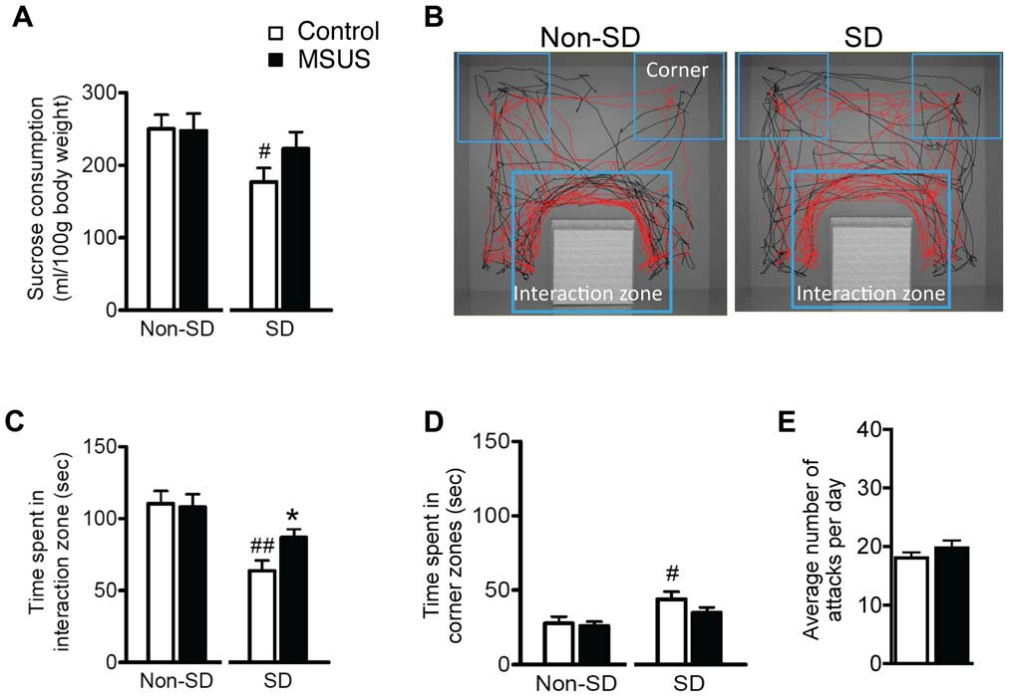
Altered response to social defeat in F2 MSUS males. **a,** Sucrose consumption in F2 MSUS and control males subjected to SD and non-SD. **b**, Images of the SD test arena showing representative tracking of position and movements in defeated (SD, right) and non-defeated (non-SD, left) animals. Interaction and corner zones are outlined with blue boxes. Trace from a control male, black. Trace from a MSUS male, red. **c,** Time in the interaction zone, and (**d**) time in corners in F2 MSUS and control males subjected to SD and non-SD. **e,** Number of daily attacks over the 2-weeks of SD in F2 control and MSUS mice. # p<0.05, SD versus non-defeated (Non-SD) F2 MSUS or control males.* p<0.05, F2 MSUS versus F2 control within SD treatment, # p<0.05, ## p<0.001 SD versus non-SD F2 MSUS or F2 control animals.

Previous work has demonstrated that rodents exposed to chronic stress show stress resilience when later subjected to aversive and stressful conditions [31], [32], [33]. We examined whether early stress results in transmissible alterations in response to social conflict by exposing F2 MSUS and control mice to chronic social defeat (SD). SD is a highly aversive form of social conflict that in rodents, impairs behavioral and neuroendocrine responses, and induces depressive symptoms such as anhedonia, strong avoidance of cues associated with defeat, stereotyped behaviors, and decreased exploratory, motivational and socio-sexual behaviors [11]. When exposed to chronic SD, control males consumed significantly less sucrose during the two weeks of SD (anhedonia) (Fig. 4a), and showed significant avoidance of odor cues from an aggressor when compared to non-SD controls (Fig. 4b–d). In contrast, F2 MSUS males showed no reduction in sucrose consumption when compared to non-SD control or MSUS males, despite exposure to a similar number of attacks (Fig. 4a, e). They also demonstrated significantly less avoidance of odor cues from an aggressor than SD control males (Fig. 4b–d).

## Discussion

The demonstration that unpredictable early stress impairs social interaction and cognition across generations, and at the same time leads to stress resilience, suggests that early stress can have both detrimental and beneficial effects on behavior, and that these effects can be inherited. The mechanisms underlying this dual effect are not known, but previous data have suggested that they likely involve epigenetic processes [9], [34]. Here, the demonstration that transmission occurs through males is important because it allows us to minimize the potential contribution of maternal care to the transmission of the effects of the manipulation. Thus, transmission of behavioral phenotypes is unlikely to result from any change in maternal care since females used for breeding with F1 MSUS males to generate F2 animals, or with F2 MSUS males to generate F3 animals, were naïve wild-type, and had normal maternal behaviors [9]. Further, since the effects were transmitted by males that did not have any contact with their pups, interference with developmental or physiological processes in the offspring is unlikely. Finally, the demonstration that the effects of early stress are observed down to F3 animals, a generation that originates from F2 sperm never exposed to stress (unlike sperm in F1 pups), strongly suggests that transmission likely occurs through the germline and does not involve environmental factors. *In vitro* fertilization experiments are under way to confirm this possibility, and exclude the contribution of anomalies in the social environment early in life. It should be noted that our previous work also demonstrated that the effects of MSUS on behavior can be transmitted through females [9], [34], suggesting a general mechanism that can involve female and male germ cells.

Interestingly, social anxiety in F2 and F3 MSUS males was not observed in F1 sires, suggesting that sires are asymptomatic but are still able to transmit the defects to their offspring. Although not understood mechanistically, the existence of asymptomatic or silent carriers has already been reported in humans [35], [36], and was previously demonstrated for other behaviors in the MSUS model [9], [34]. One possibility may be that F1 animals do not show any obvious phenotype because they were exposed to MSUS only postnatally (from birth to PND14), unlike F2 and F3 animals which carry the potential epigenetic changes starting in the fertilized egg and through both pre- and postnatal development. Alternatively, since multiple physiological and molecular changes are likely induced by early stress in F1 animals, they may in part be corrected by compensatory mechanisms, some of which may not be transmitted to the following generations. Such partial transmission may explain why specific behavioral deficits are observed in the offspring but not in F1 animals.

The transmission of social anxiety to F2 and F3 MSUS mice is sex-specific and occurs only in males, and not in females. This may be due to a sex-specificity in the mechanisms of transmission or alternatively, to differences in the expression of behavioral traits underlying social interactions in males and females. In mice, same-sex relationships between males involve a complex interplay between sociability, competition and aggression, while relationships between females involve less aggressive components.

Finally, these results suggest that mice subjected to MSUS show some resilience to the effects of chronic SD and therefore, that early stress, although impairing social behaviors under normal conditions, can also act to adapt or prepare individuals and subsequent generations to stressful environments. Such adaptation may involve serotonergic pathways, an idea that is consistent with previous evidence showing that enhanced serotonin release in DR target areas may account for the behavioral effects of SD [37]. In the present model, the marked decrease in 5HT1AR binding and increase in serotonin release may make the serotonergic system less amenable to respond to SD, and weaken its impact. Although other neurotransmitter systems including noradrenergic or dopaminergic pathways may also be involved, the data further suggest an association between altered 5HT1AR signaling and abnormal social behaviors in MSUS offspring.
